# DAG-MAG-ΒHB: A Novel Ketone Diester Modulates NLRP3 Inflammasome Activation in Microglial Cells in Response to Beta-Amyloid and Low Glucose AD-like Conditions

**DOI:** 10.3390/nu17010149

**Published:** 2024-12-31

**Authors:** Valentina Gentili, Giovanna Schiuma, Latha Nagamani Dilliraj, Silvia Beltrami, Sabrina Rizzo, Djidjell Lara, Pier Paolo Giovannini, Matteo Marti, Daria Bortolotti, Claudio Trapella, Marco Narducci, Roberta Rizzo

**Affiliations:** 1 Department of Environmental and Prevention Sciences, University of Ferrara, 44121 Ferrara, Italy; valentina.gentili@unife.it (V.G.); giovanna.schiuma@unife.it (G.S.); silvia.beltrami@unife.it (S.B.); sabrina.rizzo@unife.it (S.R.); djidjell.lara@edu.unife.it (D.L.); daria.bortolotti@unife.it (D.B.); marco.narducci@tuj.temple.edu (M.N.); 2 Department of Chemical, Pharmaceutical, Agricultural Sciences, University of Ferrara, 44121 Ferrara, Italy; latha.dilliraj@edu.unife.it (L.N.D.); gvnppl@unife.it (P.P.G.); trap@unife.it (C.T.); 3 Department of Translational Medicine, University of Ferrara, 44121 Ferrara, Italy; matteo.marti@unife.it; 4 Management Department, Temple University, Japan Campus, Tokyo 154-0004, Japan

**Keywords:** DAG-MAG-ΒHB, Alzheimer’s disease, therapeutic ketosis, blood–brain barrier, neuroprotection, ketone diester, exogenous ketones

## Abstract

Background: A neuroinflammatory disease such as Alzheimer’s disease, presents a significant challenge in neurotherapeutics, particularly due to the complex etiology and allostatic factors, referred to as CNS stressors, that accelerate the development and progression of the disease. These CNS stressors include cerebral hypo-glucose metabolism, hyperinsulinemia, mitochondrial dysfunction, oxidative stress, impairment of neuronal autophagy, hypoxic insults and neuroinflammation. This study aims to explore the efficacy and safety of DAG-MAG-ΒHB, a novel ketone diester, in mitigating these risk factors by sustaining therapeutic ketosis, independent of conventional metabolic pathways. Methods: We evaluated the intestinal absorption of DAG-MAG-ΒHB and the metabolic impact in human microglial cells. Utilizing the HMC3 human microglia cell line, we examined the compound’s effect on cellular viability, Acetyl-CoA and ATP levels, and key metabolic enzymes under hypoglycemia. Additionally, we assessed the impact of DAG-AG-ΒHB on inflammasome activation, mitochondrial activity, ROS levels, inflammation and phagocytic rates. Results: DAG-MAG-ΒHB showed a high rate of intestinal absorption and no cytotoxic effect. In vitro, DAG-MAG-ΒHB enhanced cell viability, preserved morphological integrity, and maintained elevated Acetyl-CoA and ATP levels under hypoglycemic conditions. DAG-MAG-ΒHB increased the activity of BDH1 and SCOT, indicating ATP production via a ketolytic pathway. DAG-MAG-ΒHB showed remarkable resilience against low glucose condition by inhibiting NLRP3 inflammasome activation. Conclusions: In summary, DAG-MAG-ΒHB emerges as a promising treatment for neuroinflammatory conditions. It enhances cellular health under varying metabolic states and exhibits neuroprotective properties against low glucose conditions. These attributes indicate its potential as an effective component in managing neuroinflammatory diseases, addressing their complex progression.

## 1. Introduction

Metabolic dysfunctions and neurodegenerative disorders, such as Alzheimer’s disease (AD), present complex challenges that require innovative approaches for mitigation and treatment.

Alzheimer’s disease (AD), the leading cause of dementia, is a neurodegenerative condition that progressively impairs memory and cognitive functions, eventually hindering basic tasks [1]. It represents a significant global health issue, with considerable societal and economic impacts. AD is characterized by distinct pathological features within the central nervous system (CNS), known as the hallmarks of AD. These include (i) extracellular accumulation of amyloid precursor protein (APP)-derived amyloid beta (Aβ) oligomers [2,3]; (ii) intracellular build-up of hyperphosphorylated tau proteins, leading to neurofibrillary tangles [2,3,4,5]; and (iii) the formation of dense and inflamed plaques [2,6]. The interaction of these pathologies results in synaptic damage and neuronal loss, which exacerbate a self-perpetuating feedback loop that further amplifies the pathological hallmarks and complicates therapeutic interventions [2,7]. The extent and intensity of CNS exposure to continuous stressors influences the progression and severity of AD [2,8]. These internal and external CNS stressors disrupt the blood–brain barrier (BBB) and vascular integrity while causing significant disturbances in CNS metabolism, oxygenation, and defense mechanisms, leading to mitochondrial damage that compromises CNS structural functionality [2,9]. Therefore, Aβ expression in neuronal and glial populations arises from CNS-centric stressors [7], serving as both a pathological hallmark and a marker for neuronal stress, energetic crisis, mitochondrial dysfunction, and inflammation [2]. These stressors contribute to systemic regulatory degeneration, chiefly controlling reactive oxygen species (ROS) concentrations and activities within the CNS. Therefore, the primary cause of AD is the amalgamation of multiple chronic disturbances adversely affecting CNS metabolism and CNS integrity. This hostile environment exacerbates neuronal distress, promoting Aβ upregulation. If unchecked, this distress reinforces neuropathological events, leading to neurodegeneration and AD symptoms, as suggested in recent AD biomarker evolution models [10]. Thus, in the intricate paradigm of AD management, a primary therapeutic goal is the nuanced modulation of allostasis to mitigate the key accelerants propelling the onset and progression of AD’s distinct pathophysiological features.

Since the 1930s, a large array of animal studies and placebo-controlled clinical trials have provided much evidence that the induction of acute, transient, and moderate hyperketonemia—generally referred to as nutritional ketosis (>0.5 mmol/L–<6 mmol/L)—exerts a large spectrum of neuroprotective benefits [11,12,13]. The concept of increasing blood KB in AD is gaining traction, largely due to insights from brain imaging studies. Ketone metabolism is preserved in early AD as ketones are pushed into the brain in proportion to their plasma concentration (0.02–12 mM) [12] and irrespective of glucose availability, whereas glucose is pulled into the brain in proportion to its utilization by astrocytes and neurons, a direct reflection of the rate of glycolysis. Therefore, KB has the potential to bypass the vicious cycle between gradual brain exhaustion and AD.

Exogenous ketonic compounds (EKC) have been considered alternative methods by which to elevate blood ketones without the need for strict dietary changes [14,15]. EKC includes various forms of ketone salts and ketone esters that readily deliver the most abundant ketone body β-hydroxybutyrate (βHB) into circulation. Among the EKC, the consumption of ketone esters has been shown to be a promising approach by which to induce sustained therapeutic ketosis efficiently and robustly, which is necessary to mitigate AD pathophysiology [16,17].

Nevertheless, the ingestion of ketone esters (KE) may also cause a mild to moderate range of gastrointestinal distress, including flatulence, nausea, diarrhea, vomiting, and stomach upset [18]. Additionally, over the long term, ingestion of ketone esters may overload the liver and kidney, warranting further study, particularly for those with pre-existing conditions [19]. Furthermore, there is also little research that demonstrates how KE safely traverses the blood–brain Barrier (BBB) without inducing structural alterations [20].

Therefore, there is a need to provide novel forms of exogenous ketones that address the safety and side effects highlighted above while simultaneously addressing the allosteric conditions linked to the development and exacerbation of AD. Under these premises, the primary goal of this study is to examine the safety profile of a novel neuroprotective ketone diester and both mono- and diesters of 2,3-dihydroxypropyl (R)-3-hydroxybutyrate and 2-hydroxy-1,3-propanediyl (R)-3-hydroxybutyrate (DAG-MAG ΒHB). The study will investigate the safety of DAG-MAG ΒHB across sub-chronic phases in mice, as well as its capability to traverse the blood–brain Barrier (BBB) without inducing structural alterations in vitro. Secondly, the study aims to determine if DAG-MAG ΒHB can uphold cellular bioenergetic homeostasis and therefore viability in adverse metabolic states, such as hypoglycemia. The research will examine DAG-MAG-ΒHB’s capacity to alleviate subclinical central nervous system (CNS) stressors and insults, which are considered allostatic accelerants in the onset and advancement of AD’s unique pathophysiological features. These include dyshomeostatic conditions, such as inflammasome activation, mitochondrial dysfunction, and diminished phagocytosis. The study’s findings will contribute to the understanding of DAG-MAG-ΒHB’s therapeutic potential in addressing the multifaceted nature of AD.

## 2. Materials and Methods

### 2.1. Chemical Synthesis

**Scheme 1:**
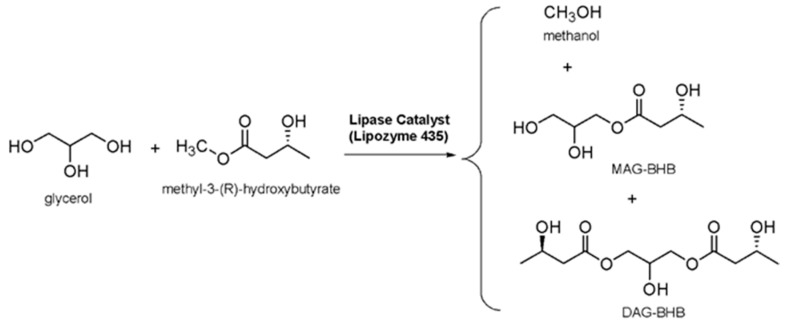
Overall reaction scheme.

DAG/MAG-ΒHB is synthesized by transesterification of glycerol with methyl (R)-3-hydroxybutyrate [21]. The reaction is catalyzed by the lipase B from Candida antarctica—CAL-B (Lipozyme 435; Novozyme, Lyngby, Denmark), and methanol is formed as the coproduct (Scheme 1: overall reaction).

**Scheme 2:**
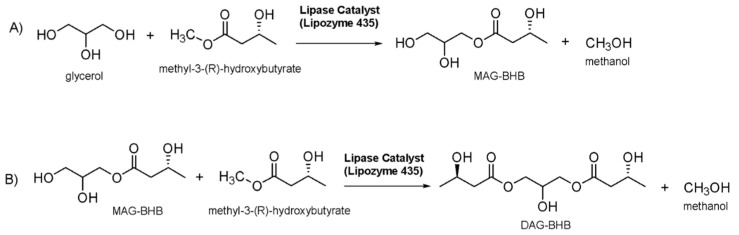
Mechanisms of (**A**) MAG- and (**B**) BAG-ΒHB formation.

The reaction mechanism involves the attack of one of the primary hydroxyl groups of the glycerol to the ester group of the methyl (R)-3-hydroxybutyrate. This step leads to the formation of the MAG-ΒHB and methanol (reaction A, Scheme 2). The methanol is continuously removed from the reaction by vacuum distillation (55 °C at 6 × 10^−1^ torr) while the MAG-ΒHB gradually replaces the glycerol as the alcoholic reagents in the attack to the ester group of the methyl (R)-3-hydroxybutyrate, leading to the formation of the DAG-ΒHB (reaction B, Scheme 2).

### 2.2. Animals

A total of 32 male and 32 female ICR (CD-1^®^) mice, 3–4 months old and weighing 25–30 g (ENVIGO Harlan Italy, Udine, Italy; bred inside the Laboratory for Preclinical Research (LARP) of the University of Ferrara, Italy) were group-housed (4 mice per cage; a floor area per animal of 80 cm^2^; a minimum enclosure height of 12 cm on a 12:12 h light–dark cycle (light on at 06:30) at a temperature of 20–22 °C and humidity of 45–55% and were provided ad libitum access to food (Diet 4RF25 GLP; Mucedola, Settimo Milanese, Milan, Italy) and water. Experiments were performed during the light phase. The experimental protocol followed in the present study was in accordance with the new European Communities Council Directive of September 2010 (2010/63/EU), a revision of Directive 86/609/EEC, and was approved by the Italian Ministry of Health (license n. 223/2021-PR and extension CBCC2.46.EXT.21) and the Ethics Committee of the University of Ferrara. According to the ARRIVE guidelines [22], all possible efforts were made to minimize the number of animals used, minimize the animals’ pain and discomfort, and reduce the number of experimental subjects. The evaluation of the concentrations was according with according to OECD guideline 408 [23], and the number of mice in each group was based on previous results of toxicity in mice models [24,25]. We obtained an alpha value of 0.05 with 8 mice per group. Mice were divided into two groups: control group (vehicle) and DAG/MAG-ΒHB treatment group for pharmacokinetics analysis and control group (vehicle) and DAG/MAG-ΒHB treatment group for toxicity, clinical and histological analyses. The only person who knew which mice were treated was the laboratory biologist responsible for the experiment. Randomization was performed by using a computer-generated random sequence to assign the experimental units to the control and treatment groups, ensuring an unbiased distribution.

Mice received a sub-chronic administration of 500 mg/kg, 1000 mg/kg, and 2000 mg/kg of DAG/MAG-ΒHB for 15 weeks, according to OECD guideline 408 [23], with a one-shot oral gavage of 4 μL/g. A saline solution of 0.9% NaCl was used as the vehicle solution.

### 2.3. Pharmacokinetics

Pharmacokinetics evaluation was performed in blood samples collected at the intervals of 30, 60, 90, and 120 min post-supplementation with 0.9% NaCl saline solution as the vehicle or DAG/MAG-ΒHB at the concentrations of 500 mg/kg, 1000 mg/kg, and 2000 mg/kg [24]. Gas chromatography was used to measure blood DAG-MAG/ΒHB.

### 2.4. Toxicity Tests

After administration, mice were observed continuously for 2 h and intermittently for up to the evaluation period, for the following toxicity parameters [25]:

Convulsions: The presence of generalized tonic–clonic seizures or focal seizures was recorded. The latency to convulsion onset and the severity of convulsions were noted using a modified Racine scale.

Myoclonus: Sudden, involuntary muscle jerks were documented by visual inspection.

Tremor: Persistent rhythmic involuntary shaking was assessed and scored on a scale of 0 to 3 (0 = absent, 3 = severe).

Hyperreflexia: Exaggerated reflex responses were evaluated using a gentle tap on the tail or limb with a reflex hammer.

Tail elevation: Tail rigidity or elevation was scored on a scale of 0 to 2 (0 = absent, 2 = sustained elevation).

Eyelid ptosis: The degree of eyelid drooping was recorded on a scale of 0 to 2 (0 = normal, 2 = completely closed).

### 2.5. Clinical Observations

After administration, mice were observed continuously for 2 h and intermittently for up to the evaluation period, for the following clinical parameters [25]:

Alteration of secretions and excretions: Excessive salivation, lacrimation, or nasal discharges were visually assessed and scored (0 = absent, 3 = severe).

Piloerection: The presence or absence of raised fur (indicative of sympathetic activation) was recorded and scored (0 = absent, 2 = prominent piloerection).

Vasodilation: Observable skin redness in the ears, paws, or tail was documented. A scoring system was employed (0 = no redness, 2 = severe redness).

Vasoconstriction: Blanching or pallor of skin, especially in the extremities, was recorded. A score of 0 to 2 was used (0 = normal, 2 = severe blanching).

### 2.6. Histological Studies

The samples of the brain, heart, spleen, liver, intestine, lung, and kidney of the two groups of animals (vehicle-treated and DAG/MAG-BHB-treated) were collected [25]. The organs of DAG/MAG-BHB- and vehicle-treated animals were immediately removed, weighed, and placed in 10% buffered formalin for 48 h, then embedded in paraffin. Paraffin-embedded tissue specimens were sectioned at 5 μm, and then haematoxylin and eosin stain were performed. For histological investigations, the sections were stained with haematoxylin and eosin and then observed under optical microscope (Nikon Eclipse E90i; Nikon, Roma, Italy).

### 2.7. In Vitro Model of Intestinal Absorption

The intestinal absorption of DAG/MAG-ΒHB was determined using an in vitro human intestinal model based on human Caco-2 intestinal adenocarcinoma cells (ATCC, HTB-37TM) and organized as a functional monolayer on Transwell^®^ inserts. Caco-2 cells were cultured in Eagle’s minimum essential medium (ATCC, No. 30-2003) with 20% fetal bovine serum and 1% penicillin/streptomycin. For oral digestion, 17.5 mL of the stock simulated salivary fluid (SSF) solution, 1.5 mL of α-amylase solution (1.3 mg/mL) (Sigma-Aldrich, St. Louis, MO, USA), 125 µL of 0.3 M CaCl_2_ and water up to 25 mL were added to each sample and placed in a 100 mL vessel [26]. The new solution was mixed for 30 min at 37 °C in darkness. For the gastric digestion, 18.75 mL of the stock simulated gastric fluid (SGF) (Table 1), 1.86 mL of pepsin solution (1 g of pepsin in 10 mL of 0.1 M HCl), 12.5 µL of 0.3 M CaCl_2_, water up to 25 mL, and 1 M HCl up to pH 3 were added. After 2 h incubation at 37 °C in darkness, intestinal digestion was performed. For this phase, 27.5 mL of the stock simulated intestinal fluid (SIF), 12.5 mL of pancreatin solution (0.008 g/mL), 6.25 mL of bile salts (0.025 g/mL), 100 µL of 0.3 M CaCl_2_, and water up to 50 mL were added. Then, the pH was raised to 7 using 1 M NaOH and incubated in a water bath at 37 °C and shaken during the process. After the in vitro simulated gastrointestinal digestion, the reactions (gastric and intestinal digestions) were stopped by freezing the samples at −18 °C and finally stored at −80 °C until lyophilization.

After the digestive process, the solution was added to the apical compartment of in vitro intestinal epithelium. Digestive fluids without DAG/MAG-ΒHB were used as the negative control, while HBSS with 1% BSA was added to the basolateral compartment. After 3 h of exposure, the apical and basolateral fractions were recovered, and DAG-MAG-ΒHB content was determined with UPLC-MS analysis. The bioavailability was calculated and expressed as a percentage of uptake (%), calculated on the mass balance of the active ingredient (amount of active ingredient in apical + amount of active ingredient in basolateral).

### 2.8. Assessment of Cell Viability

For cell viability assessment, HCM3 microglial cells (CRL-3304; ATCC) were seeded and treated when they had reached 50–60% of confluence. Twenty-four hours after the administration of DAG/MAG-ΒHB (12.5, 25, 50 mM), cell viability was examined by MTT (3-(4,5-dimethilthiazol-2yl)-2,5-diphenyl tetrazolium bromide) colorimetric assay (Roche Diagnostics Corporation, Indianapolis, IN, USA) [27].

### 2.9. Gas-Chromatograph

After a centrifugation step (2750× *g* for 5 min), DAG-MAG/ΒHB or trimethylsilyl (TMS) ether (glucose derivatizated) were quantified in the pellet (equivalent to the excreted fraction) and the supernatant (corresponding to the bioaccessible fraction) by the gas chromatographic method. A weighed amount of the analyte (0.01–4 mg) was diluted with pyridine (0.300 mL). A 0.02 mL volume of a 20% (*w*/*v*) solution of ethyl 6-hydroxyhexanoate (internal standard) in pyridine and MSTFA (0.05 mL) were added. The mixture was gently shaken at 50 °C for 2 h. After that, the mixture was diluted with pyridine (0.63 mL) and injected (0.001–0.003 mL) into the gas chromatograph. The gas chromatograph was set up in split mode (injector temperature 250 °C, split ratio 50, carrier He 1 mL/min) and equipped with an FID detector (detector temperature 300 °C) and a MEGA-S52 capillary column (30 m length × 0.32 mm internal diameter × 0.1–0.15 µm film thickness) from MEGA Snc. The chromatographic run was performed with an oven temperature program starting from 100 °C (maintained for 2 min) and growing to 280 °C, with a temperature gradient of 10 °C/min allowed so as to separate DAG/MAG-ΒHB with the retention time of 11.47 min for MAG, 19.02 for DAG-ΒHB, and 15 min for TMS.

### 2.10. Cell Cultures

Human HMC3 microglial cells (CRL-3304; ATCC) were cultured in EMEM medium, 10% heat-inactivated fetal bovine serum, 1% antibiotic–antimycotic, at 37 °C/5% CO_2_ humidified incubator. RPMI and EMEM were devoid of additives and were applied throughout inflammasome activation tests.

### 2.11. Immunoenzymatic ELISA Evaluation of Pyruvate, Lactate, Acetoacetate (AcAc), Acetyl-CoA (ACoA), SCOT and BDH1

The levels of pyruvate, lactate, acetoacetate (AcAc), acetyl-CoA (ACoA), SCOT and BDH1were assessed in cell lysates. Cells were washed twice with ice-cold PBS to remove residual media and detached using a scraper. Cell pellets were resuspended in ice-cold lysis RIPA buffer (volume: ~100 µL per 1 × 10^6^ cells), including protease inhibitors. The lysate was kept on ice for 15–30 min to ensure complete lysis. Cells were vortexed gently every 5 min to enhance cell membrane disruption. The lysate was centrifuged at 12,000–16,000× *g* for 10–20 min at 4 °C to pellet cellular debris. The clear supernatant was used for ELISA assays: acetyl-CoA (A-CoA) ELISA Kit (Elabscience, Houston, TX, USA); acetoacetate and pyruvate assay kit (Abcam, Cambridge, UK), lactate assay kit (MyBiosource, San Diego, CA, USA) human OXCT1 (SCOT) ELISA Kit (ELK Biotechnology, Wuhan, China); and BDH1 assay (MyBiosources).

### 2.12. Blood–Brain Barrier Model

The inner surface of the Transwell membranes (Corning Transwell 12-well plates) was coated with collagen (R&D Systems) to enhance cell attachment. We seeded human brain endothelial cells (hBMECs) (Innoprot, Derio, Spain) onto the apical side of the Transwell inserts at a density of approximately 100,000 cells/cm^2^, in endothelial cell medium (ECM) medium (Innoprot, Spain).

### 2.13. Priming of Inflammasomes

Inflammasome activation was measured according to Primiano et al.’s description [28]. HCM3 cells were plated in EMEM without supplements at 1 × 10^5^ cells/well. Cells were stimulated for 1 h with 10 g/mL (13.4 mM) of nigericin (tlrl-nig, InvivoGen, San Diego, CA, USA) for 2 h with 2 mM ATP (tlrl-atpl, InvivoGen), or overnight with 200 g/mL of monosodium urate crystals (MSU; tlrl-msu, InvivoGen) for NLRP3 induction; flagellin/DOTAP complex, obtained with 25 μL HBS buffer with 500 ng flagellin (final concentration at 1 μg/mL; tlrl-stfla, InvivoGen) and 3 μL DOTAP (11202375001, Sigma-Aldrich) for each well for NLRC4 induction; with 0.5 mL Opti-MEM supplemented with GlutaMax (Opti-MEM™ Reduced Serum Medium containing GlutaMAX™ Supplement, 51985034, Gibco, Grand Island, NY, USA) containing 1 μg/mL Bacillus anthracis lethal factor (172B, List Biological Laboratories, Campbell, CA, USA) for 3–6 h for NLRP1 induction; and with a poly(dA:dT)/lipofectamine complex, obtained by mixing 50 μL Opti-MEM containing 1 μg poly(dA:dT) (final concentration at 2 μg/mL) with 50 μL Opti-MEM containing 2.5 μL lipofectamine 2000 (11668027, Thermo Fisher’s Scientific, Waltham, MA, USA) for each well for AIM2 induction. HCM3 were also treated with 50 nM TLR4 inhibitor (CLI-095; InvivoGen) or primed with 1 μM of Aβ1-42 (ab120301) (Abcam) for 24 h in serum free media. Plates were centrifuged at 15,000 rpm for 5 min after the proper activation times, supernatants were collected for cytokine and caspase analyses, and cells were lysed for immunoblot analyses.

### 2.14. RNA Extraction and Retrotrascription

Using the Trizol reagent (Invitrogen, Waltham, MA, USA), total RNA was isolated from HCM3 cells. Briefly, genomic DNA contamination was removed from 2 g of RNA using RNase-free DNase I (Takara, Kusatsu, Japan) [29]. cDNA was created using the SuperScript III First Strand kit (Invitrogen) and kept at 80 °C until needed.

### 2.15. Quantitative Real-Time PCR

SYBR Green (ThermoFisher’s) and the proper primers were used to perform quantitative real-time PCR for NLRP3 expression analysis. For comparative evaluation, data were standardized to GAPDH using the 2(Ct) technique. Primers are reported in Table 2 [29,30].

### 2.16. Caspase-Glo-1 Inflammasome Assay

Caspase-1 activity was measured in the supernatants using the Caspase-Glo-1 inflammasome assay (Promega, Madison, WI, USA). Briefly, cell supernatants were combined with Z-WEHD aminoluciferin substrate, and luminescence was measured following 1 h incubation by Tecan (Tecan, Männedorf, Switzerland).

### 2.17. Western Blotting

Cells were lysed in a RIPA buffer containing a cocktail of protease inhibitors and phosphatase inhibitors [31]. Samples of 5–10 μg of total cell lysates were separated on Bio-Rad 4–12% Bis-Tris gels (Bio-Rad, Hercules, CA, USA) in MES buffer (Thermo Fisher’s Scientific) and then transferred to PVDF membranes (Merck Millipore, Burlington, MA, USA). After transfer, the membranes were blocked with 5% skim milk in TBST (Tris-buffered saline containing 0.1% Tween-20) for 1 h and incubated with primary antibodies at 4 °C overnight. After incubation, the membranes were incubated with secondary antibodies for 1 h at room temperature. Protein bands were visualized by GelDoc Western blotting detection reagents (Bio-Rad). The primary antibodies used for immunoblotting are IL-1β (1:1000, AF-401-NA, R&D Systems, Minneapolis, MN, USA), and caspase-1 (1:1000, 4199, Signaling Technology, Denver, MA, USA).

### 2.18. Immunocytochemistry

HCM3 was fixed in 4% PFA for 20 min at RT [32]. The fixed cells were then washed with PBS and permeabilized in PBS containing 0.1% Triton X-100 and 10% normal horse serum for 1 h. Cells were incubated with primary antibodies overnight at 4 °C. After washing with PBS, the cells were incubated with anti-ASC rabbit antibody (1:500, NBP1-78977, Novus) and Alexa Fluor 594-conjugated anti-rabbit IgG secondary antibody for 1 h. Cell nuclei were stained with DAPI. The images were obtained from a Nikon fluorescence microscope with a 40× objective lens. The images were processed using Nikon software (NIS Elements, Tokyo, Japan).

### 2.19. Phagocytosis Assay (Bead Uptake Assay)

Phagocytic activity was measured from HCM3 microglial cells that phagocytosed Fluoresbrite YG microspheres (Polysciences (Warrington, PA, USA), 17147). Beads were opsonized with 50% FBS in DMEM at 37 °C with shaking for 30 min before the assay. After induction of NLRP3 inflammasome and DAG-MAG/ΒHB treatment, microglia were treated with opsonized beads for 1 h in a dark cell incubator for the uptake of beads. All steps after the bead uptake were conducted on ice. Cells were washed twice with prewarmed 0.1 M PB and treated with prewarmed 0.25% trypsin for two minutes. An amount of 1 mL FACS buffer (PBS with 2% FBS and 0.5% sodium azide) was then added to inactive trypsin. The cells were collected by pipetting and centrifuged at 4 °C and 956× *g* for 5 min. Pellets were washed once with FACS buffer, centrifuged, and resuspended in 100 μL FACS buffer containing CD11b-APC antibody (eBioscience (San Diego, CA, USA), 1:1000). After incubating for 30 min at 4 °C, cells were washed twice with FACS buffer and analyzed with FACSCantoII (BD Bioscience). A total of 10,000 APC-labeled cells were read, and the cells with more than two beads were gated for analysis.

### 2.20. Mitochondrial Membrane Potential Assay

JC1 system (ThermoScientific) was used to assess active mitochondria following the producer’s instructions.

### 2.21. ATP Content Assay

ATP content was measured with ATP probes from an ATP determination kit (ThermoFisher’s) following the producer’s instructions.

### 2.22. Intracellular Reactive Oxygen Species (ROS) Assessment

Reactive oxygen species levels were estimated using the probe CM-H2DCFDA (molecular probes, Invitrogen) [33]. HCM3 cells were cultured at 6 × 10^4^ cells per well and incubated with a 10 μM CM-H2DCFDA probe for 30 min at 37 °C and washed twice in PBS. Fluorescent cells were visualized by a Nikon fluorescence microscope.

### 2.23. Statistical Analysis

Statistical analysis was performed by a parametric approach for the normal distribution, as assessed using the Kolmogorov–Smirnov test. The Student’s *t* test was used to compare variables, Fisher’s exact test was used to compare percentages. A value of *p* < 0.05 was accepted as statistically significant. The statistical analysis was performed by GraphPad software version 9.

## 3. Results

### 3.1. Chemical Characterization

**Figure 1:**
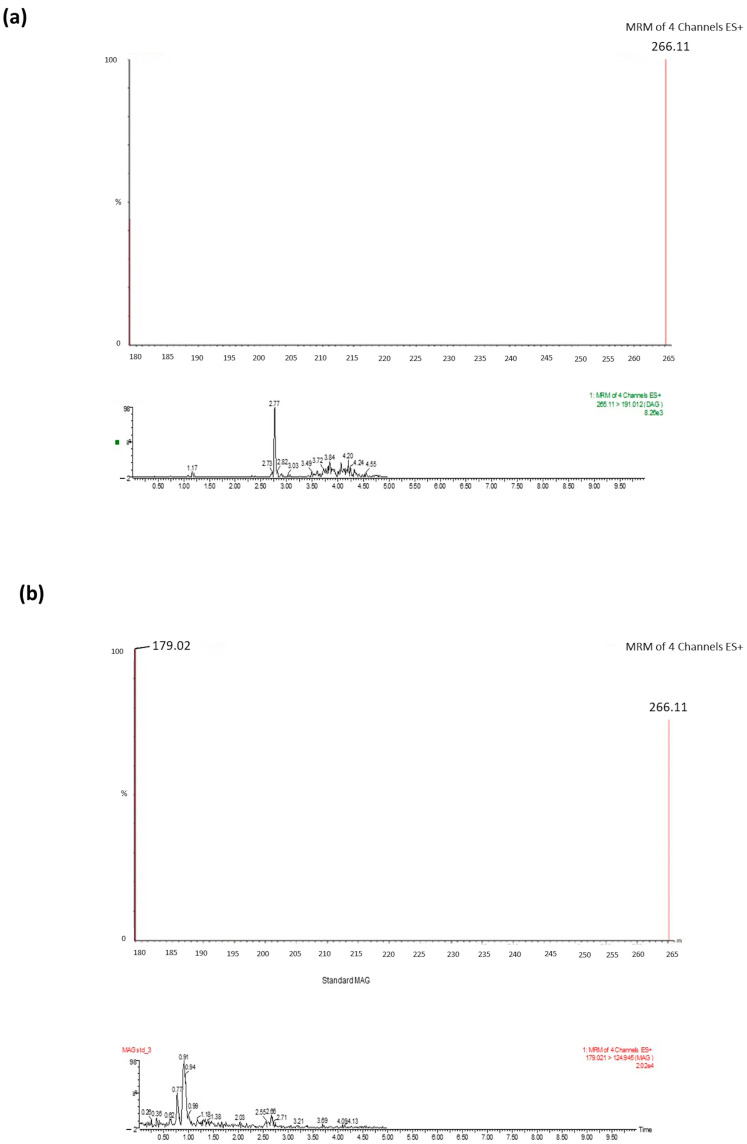
ESI-MS spectrum of (**a**) DAG-ΒHB and (**b**) MAG-ΒHB.

A sample (3 g) of the product mixture coming from a standard synthetic procedure was purified by flash column chromatography on silica gel 60 (230–400 mesh) with cyclohexane-ethyl acetate-methanol (3:5:0.5) as the eluent. The eluted fractions were analyzed TLC on silica gel 60 F254 with cyclohexane-ethyl acetate-methanol (1:7:1) as the eluent and phosphomolybdic acid as the detection agent. The fractions containing pure DAG-ΒHB (rf = 0.3) and MAG-ΒHB (rf = 0.2) were collected, and the eluent was removed under vacuum. The molecular weights of the pure compounds were determined by mass spectrometry (Figure 1a,b). The structures of the pure compounds were assigned by 1H- and 13C-NMR analysis (Supplementary Figure S1a–d). For a more accurate interpretation of the 1H-NMR spectra, the corresponding two-dimensional COSY experiments were performed (Supplementary Figure S2a,b).

### 3.2. DAG/MAG-ΒHB Bioavailability

**Figure 2:**
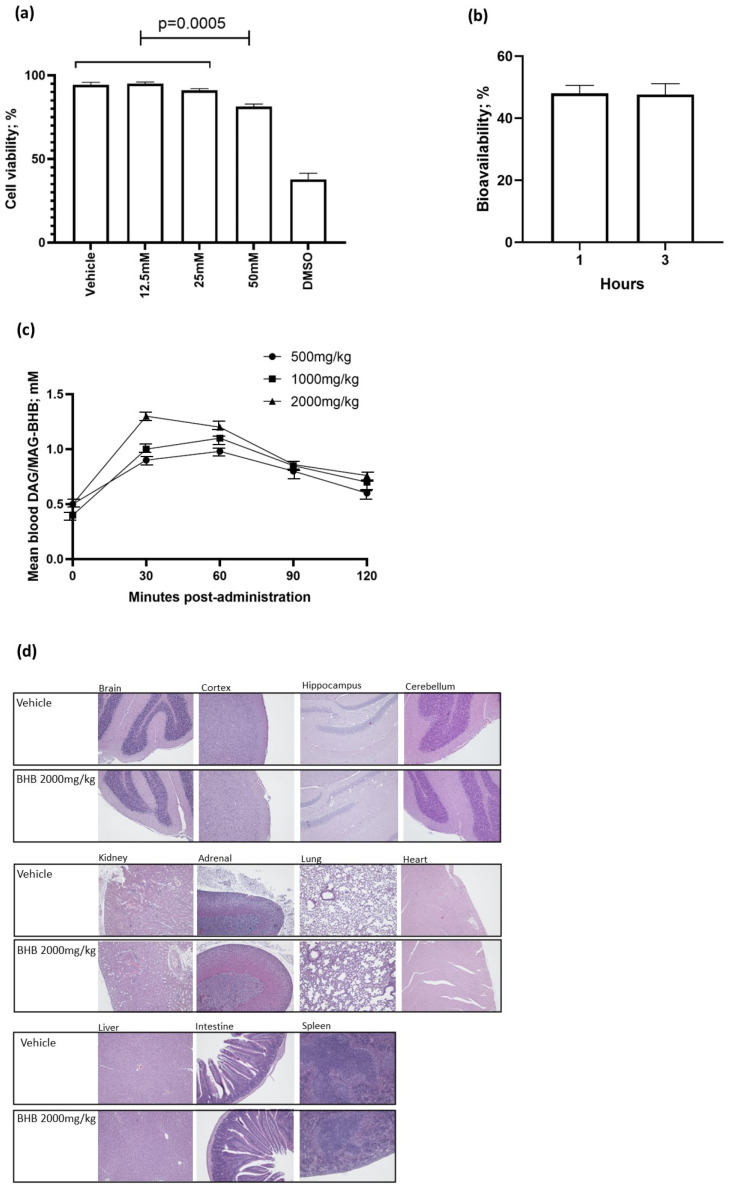
(**a**) Evaluation of cytotoxicity by MTT assay after 24 h treatment with different concentrations of DAG/MAG-ΒHB of Caco-2cells (12.5, 25, 50, 100, 150, 200 mM). (**b**) Intestinal absorption of DAG/MAG-ΒHB determined using an in vitro human intestinal model based on human Caco-2 intestinal adenocarcinoma cells. The bioavailability was expressed as a percentage of uptake (%), calculated on the mass balance of the active ingredient (amount of active ingredient in apical + amount of active ingredient in basolateral). (**c**) Pharmacokinetics evaluation was performed in blood samples collected at the intervals of 30, 60, 90, and 120 min post-supplementation with 0.9% NaCl saline solution as the vehicle or DAG/MAG-ΒHB at the concentrations of 500 mg/kg, 1000 mg/kg, and 2000 mg/kg. Gas chromatography was used to measure blood DAG-MAG/ΒHB. (**d**) Paraffin-embedded tissue specimens were sectioned at 5 μm, and then haematoxylin and eosin stain were performed. For histological investigations, the sections were stained with haematoxylin and eosin and then observed under optical microscope.

To assess DAG/MAG-ΒHB bioavailability, we analyzed intestinal absorption using a cell-based intestinal epithelium model. The digestive process was obtained by an in-vitro digestion procedure. This procedure is designed to simulate the human digestive process (divided into oral, gastric, and intestinal compartments) and is based on the use of fluids simulating the physiological secretions typical of the digestive process (saliva, gastric juices, pancreatic juices, and bile). At the end of the digestive process, the concentration of DAG/MAG-ΒHB was measured in complete digests and compared with the declared titer to estimate the overall recovery of the process. DAG/MAG-ΒHB was used at 12.5, 25, 50, 100, 150, 200 mM to assess the impact on intestinal epithelial cell viability. We observed a decrease in cell viability at 50, 100, 150 and 200 mM (*p* = 0.0005; Student’s *t* test) and a slight decrease at 25 mM (Figure 2a), so we used a concentration of 12.5 mM for the intestinal bioavailability evaluation. The intestinal absorption of DAG/MAG-ΒHB (12.5 mM) remains constant over 3 h, reaching a constant bioavailability of 50% after gastric and intestinal digestion (*p* = 0.16; Student’s *t*-test) (Figure 2b). The subsequent in vitro experiments were performed after gastric and intestinal digestion of DAG/MAG-ΒHB to simulate the process that an exogenous ketone supplementation would undergo.

### 3.3. DAG/MAG-ΒHB Pharmacokinetics and Toxicological Evaluation

The mice were administered with a dosage of 500, 1000, and 2000 mg/kg of DAG/MAG-ΒHB by oral gavage, according to EFSA OECD guideline 408, which ensures that selected doses are sufficient to elicit observable biological effects without inducing significant toxicity. To further substantiate our dose selection rationale, the in vitro viability data from Caco-2 cells (Figure 2a) provided a reassuring reference, showing no toxicity at 12.5 mM, which corresponds to an estimated in vivo equivalent of 3894 mg/kg based on molecular weight (264 g/mole) and density (1.18) calculations. This equivalence underscores the safety margin inherent in the use of 2000 mg/kg as the highest dose in our in vivo study, reinforcing its appropriateness and alignment with established safety parameters. Pharmacokinetics in the whole blood was measured post-supplementation at intervals of 30, 60, 90, and 120 min. We observed that the absorption of DAG-MAG-ΒHB reached a peak 30 min post-administration, with a level of 1–1.3 mM, that is maintained until 60 min post-administration (Figure 2c).

The mice were then visually evaluated for the presence of toxic manifestations (convulsions, myoclonus, tremor, hyperreflexia, tail elevation, eyelid ptosis), and clinical (alteration of secretions and excretions, piloerection, vasodilation, vasoconstriction) observations during a sub-chronic treatment with DAG/MAG-ΒHB (500 mg/kg; 1000 mg/kg; 2000 mg/kg). No significant signs of toxicity and clinical observations were observed in either the animals treated with the vehicle or in animals treated with DAG/MAG-ΒHB (500 mg/kg; 1000 mg/kg; 2000 mg/kg). At termination, all of the animals (100%) survived in treated mice and vehicle controls.

### 3.4. Histopathological Observations

The evaluation of the histopathological characteristics of the treated mice showed no alteration induced by DAG/MAG-ΒHB in comparison with vehicle treatment. Figure 2d reports the histopathological observations of the brain, heart, spleen, liver, intestine, lung, and kidney of the two groups of animals, vehicle-treated and 2000 mg/kg DAG/MAG-ΒHB-treated. No evident histological modification was reported.

### 3.5. DAG/MAG-BHB Preserved Human Microglial Cells in Low and High Glucose Conditions

**Figure 3:**
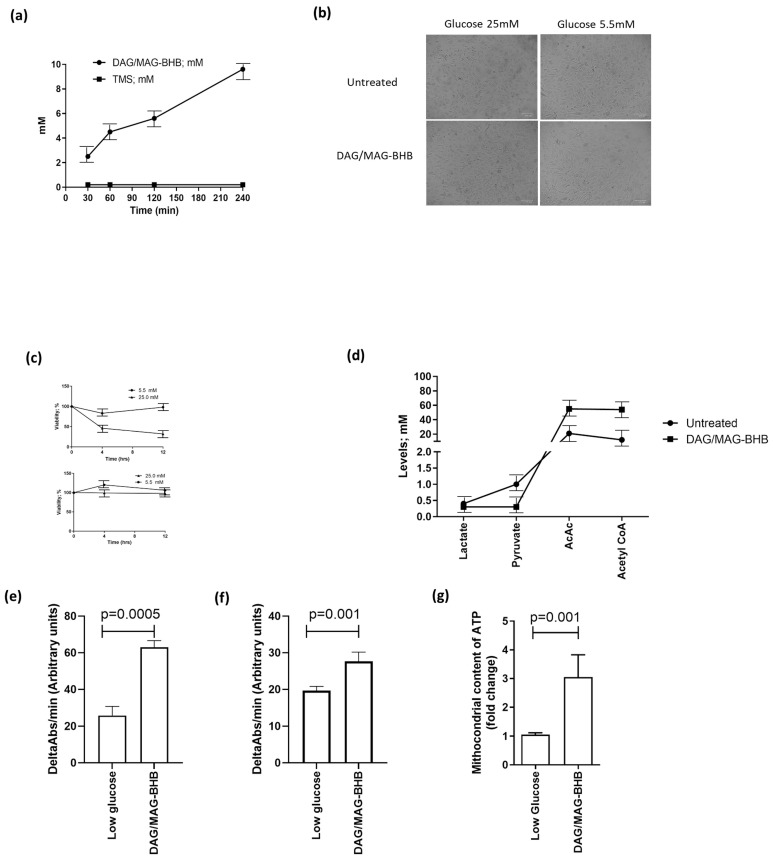
(**a**) Assessment of BBB passage of DAG/MAG-BHB (10 mM) and TMS (10 mM) through BBB in vitro system (Transwell endothelial cells (hBMECs) system) during 240 min observation. Gas chromatography was used to measure blood DAG-MAG/ΒHB and TMS. (**b**) Morphological evaluation of HMC3 cell line treated with different concentrations of glucose in the presence/absence of DAG/MAG-BHB. (**c**) Viability evaluation by MTT assay of HMC3 cell line treated with different concentrations of glucose in the presence/absence of DAG/MAG-BHB. (**d**) Levels of pyruvate, lactate, AcAc and acetylCoA in the presence of 5 mM glucose for 18 h in HMC3 cells. (**e**) BDH1, (**f**) SCOT, (**g**) ATP. Mean ± SEM for each group is reported. *p* values were obtained by statistical analysis with the Student’s *t*-test.

We applied digested DAG/MAG-ΒHB to an in vitro BBB model in order to assess the ability to pass the barrier. We observed that, in a time-lapse of 240 min, DAG/MAG-BHB showed a gradual passage through the brain barrier, with a peak at 240 min after the addition of the compound to the cell culture (Figure 3a). Because DAG/MAG BHB is able to cross the BBB and has no toxic effect in in vivo condition, we assessed the effect on human microglial cells (HMC3) in the presence of different concentrations of glucose (5.5, 25.0 mM), to mimic hypo and normal glucose. This cell line retains the key functional characteristics of primary microglia, including the ability to respond to inflammatory stimuli such as amyloid-beta and low-glucose conditions [34]. The low glucose untreated samples showed a clear modification in cell morphology (Figure 3b). On the contrary, DAG/MAG-ΒHB treatment was also able to preserve cell morphology in low glucose concentrations (Figure 3b). These results were confirmed by the viability assessment (Figure 3c), where DAG/MAG-ΒHB-treated cells maintained a high viability in comparison with untreated cells (*p* = 0.0005; student’s *t* test), at low glucose concentrations. Glucose is an essential component in cell culture media, serving as the primary energy source for cells to maintain viability and normal morphology. Without adequate glucose levels, cells may experience energy depletion, leading to impaired cellular functions, altered morphology, and ultimately cell death. The ability of DAG/MAG-ΒHB to preserve cell morphology and viability suggests that it might sustain an alternative metabolism and energy dynamic. To assess the energy dynamic in untreated and treated cells, we evaluated the production of some end products of cell metabolism, such as (i) pyruvate, the end product of the aerobic glycolytic pathway and which is crucial in oxidative phosphorylation for the production of energy-rich molecules such as NADH and FADH_2_ in aerobic condition; (ii) lactate formed from pyruvate through the reduction of pyruvate by NADH and which is essential as an alternative pathway to the regeneration of NAD^+^ from NADH in anaerobic conditions; (iii) acetoacetate (AcAc), a critical step in ketone body metabolism; (iv) acetyl-CoA (ACoA), a central molecule in cellular metabolism, serving as a key intermediate in the production of energy through the tricarboxylic acid (TCA) cycle and as a precursor for various biosynthetic processes, including fatty acid synthesis. We evaluated the levels of these molecules in the presence of low glucose conditions (5.5 mM) for 18 h. We observed low levels of lactate and increased pyruvate in both untreated and DAG/MAG-BHB-treated cells, suggesting an aerobic metabolism (Figure 3d). Interestingly, microglial cells treated with DAG/MAG-ΒHB showed higher AcAc (*p* = 0.02; Student’s *t* test) and acetylCoA (*p* = 0.017; Student’s *t* test) levels even in the presence of lower pyruvate synthesis (*p* = 0.024; Student’s *t* test). As the production of AcAc and acetylCoA from BHB is controlled by two specific enzymes, beta-hydroxybutyrate dehydrogenase 1 (BDH1) and succinyl-CoA:3-ketoacid CoA transferase (SCOT), respectively, we assessed their levels of activation. We observed a significant increase in both BDH1 (*p* = 0.0005; Student’s *t* test) (Figure 3e) and SCOT (*p* = 0.001, Student’s *t* test) (Figure 3f) activation in DAG/MAG-ΒHB-treated cells. As the increased levels of acetylCoA might correspond to an increased ATP production by mitochondria, we assessed ATP production in the different experimental conditions. DAG/MAG-BHB treatment induced higher levels of ATP production even in low glucose conditions (*p* < 0.01; Student’s *t* test) (Figure 3g), supporting the ability of DAG/MAG-BHB to also obtain a higher ATP production in a low-glucose environment.

### 3.6. DAG/MAG-ΒHB Control Aβ and Low Glucose-Dependent NLRP3 Activation in Human Microglial Cells

**Figure 4:**
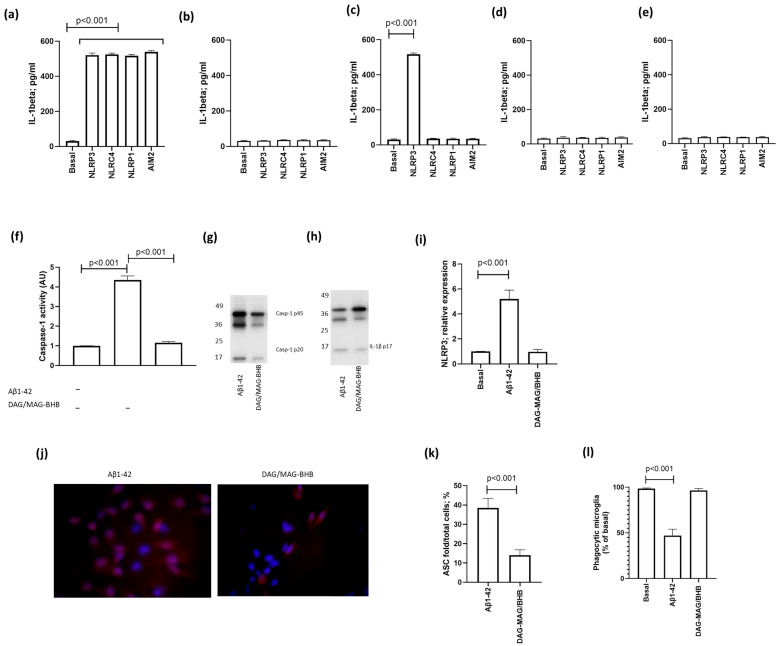
(**a**) Evaluation of IL-1beta secretion in inflammasomes-primed HCM3 cells. Evaluation of IL-1beta secretion in Aβ1-42-primed HCM3 cells in (**b**) normal (25 mM) glucose medium and in (**c**) low (5.5 mM) glucose medium. (**d**) Evaluation of IL-1beta secretion in Aβ1-42-primed HCM3 cells in low glucose medium with pharmacological inhibition of TLR4 by CLI-095. (**e**) Evaluation of IL-1beta secretion in Aβ1-42-primed HCM3 cells in low glucose medium with 10 mM DAG/MAG-ΒHB (DAG/MAG-ΒHB). (**f**) Caspase-1 activation in Aβ1-42-primed HCM3 cells in low glucose medium with or without 10 mM DAG/MAG-ΒHB (DAG/MAG-ΒHB). (**g**) Caspase-1 and (**h**) IL-1beta cleavage in Aβ42-primed HCM3 in low glucose medium in the absence (Aβ1-42) or presence of 10 mM DAG/MAG-ΒHB (DAG/MAG-BHB). (**i**) NLRP3 mRNA expression of in Aβ42-primed HCM3 in low glucose medium in the absence (Aβ1-42) or presence of 10 mM DAG/MAG-ΒHB (DAG/MAG-BHB). (**j**) Immunofluorescence staining for ASC (red) speck and nuclei (DAPI) in Aβ42-primed HCM3 in low glucose medium in the absence (Aβ1-42) or presence of 10 mM DAG/MAG-ΒHB (DAG/MAG-BHB). Scale bar: 20 μm. (**k**) Percentages of microglia containing ASC foci were quantified. (**l**) Relative phagocytic activity of microglia measured by bead uptake assay in Aβ42-primed HCM3 in the absence (Aβ1-42) or presence of 10 mM DAG/MAG-ΒHB (DAG/MAG-BHB). Mean ± SEM for each group is reported. *p* values were obtained by statistical analysis with the Student’s *t*-test and Fisher’s exact test.

Low glucose levels have been implicated in increasing neuronal vulnerability to oxidative stress and inflammation, further aggravating the neurodegenerative processes in the brain. When glucose levels plummet, such as during fasting or metabolic dysfunction, microglia may undergo metabolic reprogramming, leading to heightened inflammasome activation enabling Aβ deposition. We assessed whether Aβ and low-glucose conditions were able to modify inflammasome activation in microglial cells. We first assessed the activation of NACHT, LRR, and PYD domains-containing protein 3 (NLRP3), NLR family CARD domain-containing protein 4 (NLRC4), NLR family pyrin domain-containing protein 1 (NLRP1), and absent in melanoma 2 (AIM2), which belong to a family of intracellular pattern recognition receptors known as NOD-like receptors (NLRs), which play crucial roles in the innate immune system by sensing various pathogen- or danger-associated molecular patterns. Activation of these inflammasomes triggers caspase-1-mediated proteolytic processing and secretion of pro-inflammatory cytokines, contributing to host defense against pathogens and inflammatory responses to cellular damage. NLRP3, NLRC4, NLRP1, and AIM2 inducers were used to treat microglial cells, and IL-1beta was used as a marker of activation. Microglia cells showed an increased secretion of IL-1beta after the activation of all of the tested inflammasomes (*p* < 0.001; Student’s *t* test) (Figure 4a). Amyloid-beta (Aβ1-42)-primed microglial cells showed no inflammasome activation in normal glucose (25 mM) medium (Figure 4b), while the microglial cells showed the activation of NLRP3 inflammasome in low glucose (5.5 mM) media (*p* < 0.001; Student’s *t* test) (Figure 4c). As it has been suggested that Aβ1-42-induced NLRP3 inflammasome activation is sustained by TLR4, we performed a pharmacological inhibition of TLR4 by CLI-095. This treatment abolished Aβ1-42-induced NLRP3 inflammasome activation in low glucose conditions (Figure 4d). When we treated the Aβ1-42-primed microglial cells maintained in low glucose condition with DAG/MAG-BHB, we observed no induction of NLRP3 inflammasome activation, similar to TLR4 inhibition (Figure 4e). Similar to this, immunoblotting demonstrated that Aβ1-42-priming and low glucose increase the level of active caspase-1 (p20) (*p* < 0.001; Student’s *t* test) (Figure 4f,g) and IL-1beta (IL-1beta p17) (*p* < 0.001; Student’s *t* test) (Figure 4h). In addition, low glucose dramatically raised the levels of NLRP3 mRNA in Aβ1-42-primed microglia (*p* < 0.001; Student’s *t* test) (Figure 4i). In response to the accumulation of amyloid-beta plaques, microglia can activate the inflammasome, leading to the formation of ASC specks and subsequent release of pro-inflammatory cytokines, such as IL-1beta. While this inflammatory response initially aims to clear the amyloid-beta deposits, chronic activation of microglia and sustained release of inflammatory mediators can contribute to neurotoxicity and synaptic dysfunction, exacerbating neuronal damage. According to an immunofluorescence examination, Aβ1-42-priming and low glucose induced the creation of ASC specks, as revealed by the increased fluorescence in microglial cells (Figure 4j,k). The formation of ASC specks was significantly reduced by DAG/MAG-ΒHB treatment (*p* < 0.001; Fisher’s exact test) (Figure 4j,k). NLRP3 inflammasome activation also impacts on microglia’s phagocytic activity, essential for the maintenance of the correct health status in the brain. Aβ1-42-priming and low glucose conditions are able to reduce phagocytic activity, as shown in Figure 4l (*p* = 0.001; Fisher’s exact test), where HCM3 displayed decreased phagocytic ability in bead uptake experiments. DAG/MAG-ΒHB addition corrected this decline in phagocytic activity returning to the condition of basal phagocytosis (Figure 4l). These findings point to a direct involvement of DAG-MAG/ΒHB treatment in NLRP3 activation in AD brain.

### 3.7. DAG/MAG-ΒHB Controls NLRP3 Inflammasome Activation Maintaining Mitochondria Stabilization

**Figure 5:**
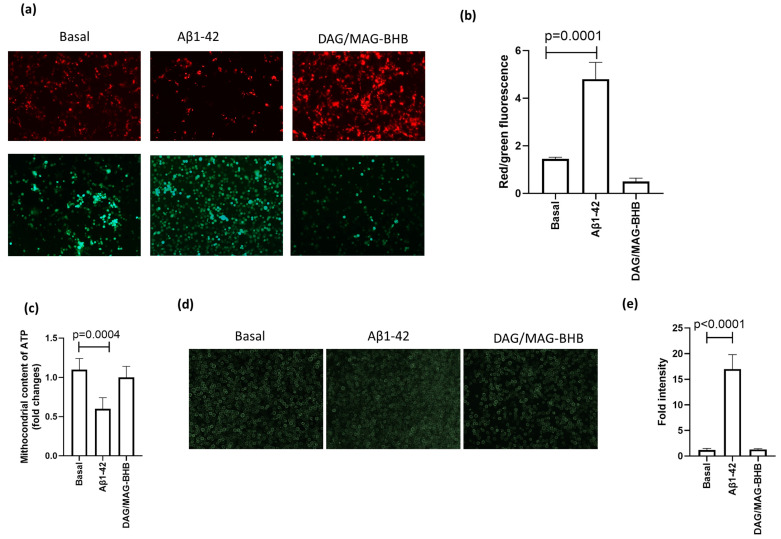
(**a**) Mitochondrial membrane potential with Jc-1 staining method in Aβ1-42-primed HCM3 in low glucose medium. (**b**) Quantitative analysis of the ratio of red/green fluorescent intensity. (**c**) ATP content in in Aβ1-42-primed HCM3 in low glucose medium. (**d**) ROS levels in Aβ1-42-primed HCM3 in low glucose medium evaluated by the probe CM-H2DCFDA. (**e**) Quantitative analysis of fold intensity of CM-H2DCFDA intensity. Mean ± SEM for each group is reported. *p* values were obtained by statistical analysis with the Student’s *t*-test.

NLRP3 activation might influence mitochondrial activity, as recent studies have unveiled the pivotal roles of mitochondria in the initiation and regulation of NLRP3. We evaluated the mitochondrial activity in HCM3 treated Aβ1-42 and maintained in low glucose medium. We observed that the mitochondrial membrane potential was reduced by Aβ1-42 priming, and DAG/MAG-ΒHB treatment restored the basal condition (*p* = 0.0001; Student’s *t* test) (Figure 5a,b). The modification in mitochondrial membrane potential reduced ATP mitochondrial content (*p* = 0.0004; Student’s *t* test) (Figure 5c), and DAG/MAG-ΒHB treatment restored the basal condition (Figure 5c). As a proof of concept of mitochondrial stabilization, we evaluated ROS generation, which has been suggested to be an absolute requirement for activation of the NLRP3 inflammasome [35]. We observed that Aβ1-42 priming increased ROS levels in HCM3 cells (*p* < 0.0001; Student’s *t* test) (Figure 5d,e), and DAG/MAG-ΒHB treatment was able to restore basal ROS levels even in the presence of an NLRP3 inducer (Figure 5d,e).

## 4. Discussion

In this study, we demonstrated that DAG/MAG-BHB is able to control the activation of NLRP3 inflammasome in response to amyloid-beta and low glucose. We showed that DAG-MAG-ΒHB is a non-toxic and safe nutraceutical compound that does not cause any morphological perturbations or loss of structural integrity. These findings align with previous research [36,37], which has focused on the relationship between ketone bodies and cell integrity. This is particularly relevant as numerous new therapeutic agents have failed in clinical trials, primarily due to their inability to preserve cell functions. This feature of DAG-MAG-ΒHB is particularly relevant for AD, as many current treatments carry a risk of side effects, making safety a paramount concern [38].

DAG-MAG-ΒHB evidenced a continuing and predictable intestinal absorption, one that ensures consistent therapeutic concentration of ketone bodies, which is essential for managing a chronic condition like AD [39].

To further deepen our understanding of the safety profile of DAG-MAG-ΒHB, we extended our assessment to its effects under varying metabolic conditions, utilizing the HMC3 human microglia cell line. Our findings indicate that DAG-MAG-ΒHB positively impacts the resident immune cells of the brain: it maintains higher viability and preserves morphological integrity, while sustaining elevated levels of acetyl-CoA and ATP. This beneficial effect was observed under low glucose concentrations. Notably, the cells treated with DAG-MAG-ΒHB exhibited significantly better outcomes when compared with the untreated cells, indicating DAG-MAG-ΒHB’s potential as a safe compound under diverse metabolic states. In fact, DAG-MAG-ΒHB effectively preserved cellular bioenergetics and structural functionality under hypoglycemia. This aligns with previous findings [39], which are the result of research on the neuroprotective effects of ketones across various metabolic states. Such work supports the idea that DAG-MAG-ΒHB can maintain cell health despite fluctuations in glucose levels, which is relevant because AD has been increasingly recognized as a condition intricately linked to a steady decline in cellular bioenergetics due to dysfunctional metabolic disturbances [40]. Hypoglycemia can also acutely impair cognitive function and contribute to hippocampal atrophy and memory dysfunction, early harbingers of AD etiology [41]. DAG-MAG-ΒHB’s ability to enhance acetyl-CoA levels independent of glucose metabolism suggests its unique value in circumventing this metabolic shortfall, which is typically reported in AD etiology. In our study, the DAG-MAG-ΒHB notably increased AcAc and acetyl-CoA levels in cells, relative to those that were untreated, despite having lower levels of pyruvate. This indicates that DAG-MAG-ΒHB may enhance acetyl-CoA production through a pathway that does not depend on glucose altering brain cell fuel preference.

To conclusively demonstrate that DAG-MAG-ΒHB utilizes an independent ketolytic pathway for acetyl-CoA production, our research focused on the interaction between DAG-MAG-ΒHB and key metabolic enzymes involved in ketone body metabolism: D-beta-hydroxybutyrate dehydrogenase (BDH1) and succinyl-CoA:3-ketoacid CoA transferase (SCOT). BDH1, a mitochondrial enzyme, plays a crucial role in the interconversion of acetoacetate and (R)-3-hydroxybutyrate, the primary ketone bodies generated during fatty acid catabolism. In contrast, SCOT is essential for ketone body utilization within the Krebs cycle, facilitating the conversion of acetoacetate into acetyl-CoA. By examining the activities of these enzymes, we aimed to elucidate the biochemical pathways through which DAG-MAG-ΒHB influences acetyl-CoA production, independent of glucose metabolism. Our research underscores the significance of our findings: DAG-MAG-ΒHB treatment substantially boosted the activity of both BDH1 and SCOT enzymes. This implies that DAG-MAG-ΒHB can effectively generate ATP through the ketolytic pathway, regardless of the presence of glucose or pyruvate. Notably, our observations reveal a remarkable increase in mitochondrial activity and ATP production following DAG-MAG-ΒHB treatment. Even under low glucose conditions, DAG-MAG-ΒHB-treated cells exhibited a significant enhancement in these mitochondrial and ATP-related parameters compared with untreated cells.

Previous publications show that ΒHB could inhibit neuronal stress by modulating multiple molecular pathways, including NLRP3-inflammosome [42,43]. More specifically, it was found that βHB attenuated AD pathology by inhibiting NLP3 inflammasome in the 5XFAD mouse model of AD [44]. In this study, we also found that DAG-MAG-ΒHB inhibited NLRP3 inflammasome. In fact, Aβ-primed microglia cells maintained in low glucose conditions showed higher levels of IL-1beta and caspase-1 activation in comparison with cells treated with DAG-MAG-ΒHB. Similarly, NLRP3 and the formation of apoptosis-associated speck-like protein containing a CARD (ASC) specks were reduced in DAG-MAG-ΒHB-treated microglial cells. The findings of this study provide compelling evidence that DAG-MAG-βHB impairs the assembly of the NLRP3 inflammasome, a key driver of neuroinflammation in Alzheimer’s disease. Immunofluorescence analysis revealed a significant reduction in the formation of ASC specks, a hallmark of NLRP3 inflammasome activation, in microglial cells treated with DAG-MAG-βHB under Aβ-primed and low-glucose conditions. ASC specks are critical for the assembly of the inflammasome, serving as a scaffold for the recruitment and activation of pro-caspase-1. By diminishing the formation of these structures, DAG-MAG-βHB disrupts the maturation and activation of the inflammasome complex, as corroborated by the decreased levels of active caspase-1 and IL-1β observed in treated cells. These results suggest that DAG-MAG-βHB interferes with the upstream assembly of the inflammasome, rather than solely acting on downstream effector pathways, thus providing a mechanistic basis for its anti-inflammatory effects in neurodegenerative contexts. This mechanistic insight underscores the potential of DAG-MAG-βHB as a therapeutic agent for conditions characterized by inflammasome-driven inflammation. This observation indicates that DAG-MAG-ΒHB treatment can effectively reverse the harmful effects caused by Aβ1−42. It is well-established that the activation of the NLRP3 inflammasome by Aβ1-42 involves the recognition of Aβ1-42 by toll-Like receptor 4 (TLR4) in microglial cells. This interaction is a significant contributor to neuroinflammation. The effect on NLRP3 and TLR4 inhibition resembles the treatment with DAG/MAG-BHB, inhibiting the inflammatory condition induced by the combination of Aβ1-42 and low glucose, and supporting the therapeutic potential of DAG-MAG-ΒHB in neuroinflammatory conditions. The mechanism by which DAG-MAG-βHB protects against Aβ toxicity aligns with prior studies, demonstrating the neuroprotective effects of βHB through indirect pathways, including the reduction of ROS and mitochondrial stabilization. Aβ toxicity is known to exacerbate oxidative stress and mitochondrial dysfunction, which contributes to neuronal damage and neuroinflammation. In our study, DAG-MAG-βHB mitigated these effects by reducing ROS levels, restoring mitochondrial membrane potential, and preserving ATP production under Aβ-primed, low-glucose conditions. These findings are supported by previous research highlighting βHB’s role in stabilizing mitochondrial function and reducing oxidative stress. For instance, Newman et al. [45] showed that βHB enhances mitochondrial efficiency and neuronal health, while Kashiwaya et al. [46] demonstrated its ability to sustain ATP production under hypoxic conditions. Moreover, βHB has been reported to inhibit the NLRP3 inflammasome, a key mediator of Aβ-induced neuroinflammation, as shown by Youm et al. [43]. These anti-inflammatory effects, coupled with the ability to bypass dysfunctional glycolytic pathways in Alzheimer’s disease models, were further emphasized by Veech et al. [47]. The reduction of ROS and mitochondrial dysfunction in Alzheimer’s models, as demonstrated by Nagpal et al. [48], underscores the relevance of βHB in counteracting Aβ-induced cellular stress. Collectively, these findings suggest that DAG-MAG-βHB operates via similar mechanisms, enhancing mitochondrial resilience and reducing oxidative stress, thereby offering robust protection against Aβ toxicity and supporting its potential as a therapeutic agent in Alzheimer’s disease.

These findings are highly significant and align with previous research which highlights how ΒHB inhibits inflammasome activation in order to attenuate Alzheimer’s disease pathology [43]. In particular, it was shown that beta-hydroxybutyrate (βHB) diminishes the activation of NLRP3 inflammasome and reduces endoplasmic reticulum (ER) stress. This reduction is mediated through the AMP-activated protein kinase (AMPK) pathway and Forkhead Box O3 (FOXO3) signaling, as observed in both live rats (in vivo) and hepatoma HepG2 cells cultured in laboratories (in vitro). Similarly, Trotta et al., in 2016 [49], found that βHB possesses anti-inflammatory properties and reduces ER stress in diabetic retinal cells. Further studies have also demonstrated the efficacy of exogenous βHB in reducing plaque formation, microgliosis, and the formation of apoptosis-associated speck-like protein containing a caspase recruitment domain (ASC) speck, as well as caspase-1 activation in the 5XFAD mouse model of Alzheimer’s disease (AD). These outcomes highlight the potential of βHB in mitigating key pathological processes associated with AD and other neurodegenerative diseases.

When we looked at mitochondrial activity in HCM3 NLRP3 inducer-treated cells, we observed that the mitochondrial membrane potential was enhanced by DAG-MAG-ΒHB treatment and ATP content was preserved, even in the presence of an NLRP3 activator. These data suggest an impact of DAG-MAG-ΒHB treatment on mitochondrial stabilization, as well as in the presence of inflammatory stimuli. In this study, we have shown that DAG-MAG-ΒHB preserved microglial cell function and integrity even in the presence of Aβ1-42. In fact, as a proof of concept of mitochondrial stabilization, we evaluated reactive oxygen species (ROS) generation, which has been suggested to be an absolute requirement for activation of the NLRP3 inflammasome [34,49,50]. We observed that DAG-MAG-ΒHB was able to maintain basal ROS levels even in the presence of an NLRP3 activator. These data support the effect of DAG-MAG-ΒHB in controlling neuronal stress, preserving microglial cell steady state even in the presence of Aβ42. We suggest that nervous system diseases involving a microglial-mediated inflammatory cascade will be susceptible to DAG-MAG-ΒHB regulation. This might be related to the ability of exogenous βHB to control inflammasome activation and sustain mitochondrial activity [51]. Mitochondrial dysfunction is a well-known feature of AD, and DAG-MAG-ΒHB’s ability to stabilize mitochondrial function and enhance energy production is an asset when addressing this aspect of AD pathology. Phagocytosis, essential for degrading misfolded and toxic proteins, plays a crucial role in preventing neurodegenerative diseases, such as AD [52]. Our study presents novel findings, including the observation that DAG-MAG-ΒHB effectively restores basal phagocytic rates in both cortical neurons and microglial cells. This restoration is particularly significant following their compromise under low glucose conditions. These results align with prior research [53], which have similarly demonstrated ΒHB’s effectiveness when inducing phagocytosis in neuronal cells. Because phagocytosis plays a crucial role in the clearance of amyloid-beta plaques and tau tangles in AD, DAG-MAG-ΒHB’s ability to modulate autophagy offers a promising therapeutic mechanism for reducing the accumulation of these pathological proteins in AD.

While this study provides significant insights into the neuroprotective effects of DAG-MAG-βHB, it is not without limitations. The individual contributions of DAG-βHB and MAG-βHB were not isolated, as the focus was on evaluating the combined efficacy of the diester/monoester formulation. Future studies should explore comparative analyses of their distinct roles in metabolic and therapeutic effects. The absence of a direct comparison with known AD compounds limits the contextualization of DAG-MAG-βHB’s efficacy relative to established therapeutics. While our study focuses on demonstrating the unique properties of DAG-MAG-βHB, including its ability to sustain energy metabolism and inhibit NLRP3 inflammasome activation, future research should include such comparisons to benchmark its therapeutic potential. Although our in vitro findings in HMC3 cells and blood–brain barrier (BBB) models robustly demonstrate neuroprotection and inflammasome modulation, we recognize the lack of more validation models. While immortalized cell lines like HCM3 offer invaluable tools for studying microglial behavior under controlled conditions, their limitations, including potential differences in gene expression, metabolic activity, and responsiveness when compared with primary cells, are well-recognized. These differences may arise from the adaptation to in vitro conditions or to the genetic modifications used for immortalization, potentially affecting the translatability of findings to in vivo contexts. To address these concerns, future studies could incorporate primary microglial cells or advanced organoid systems that more closely recapitulate the native microenvironment of the human brain. Such models provide a more physiologically relevant platform to validate findings, enabling a deeper understanding of microglial responses to stimuli such as amyloid-beta and low-glucose conditions, and offering insights into neuroinflammatory processes in a more biologically representative context. In vivo study on a mouse model was performed to assess the bioavailability and safety of DAG-MAG-βHB in complex biological systems. Further in vivo experiments might support the translational potential for AD treatment. These limitations underscore the need for future investigations to expand upon the promising in vitro and in vivo findings reported here.

## 5. Conclusions

This study provides compelling evidence for the possible therapeutic utility of DAG-MAG-ΒHB in the context of neurological disorders, particularly AD. The key strength of DAG-MAG-ΒHB lies in its ability to maintain therapeutic levels of beta-hydroxybutyrate (ΒHB), independent of the fluctuations in glucose levels. This property, combined with its non-toxic nature, positions it as a promising candidate for AD treatment. DAG-MAG-ΒHB exhibits a remarkable ability to improve cellular bioenergetics in various metabolic states, a crucial factor in managing neurodegenerative diseases. Its efficacy extends to modulating neuroinflammatory pathways, particularly by influencing the NLRP3 inflammasome, a pathway intimately linked with neurodegenerative processes. Moreover, DAG-MAG-ΒHB’s protective effects under low glucose conditions, a common challenge in AD, highlight its potential as a versatile and effective agent in neurodegenerative disease management. This study, therefore, marks an important step forward in identifying new, safe, and potent therapeutic options for combating AD and potentially other neurological disorders, paving the way for further clinical exploration and development.

While this study provides valuable insights into the neuroprotective effects of DAG-MAG-βHB, several avenues for future research remain. One key direction is the isolation and characterization of the individual contributions of DAG-βHB and MAG-βHB. By performing comparative analyses of their metabolic and therapeutic effects, future studies could delineate whether their combined efficacy arises from synergistic interactions or if one ester exhibits predominant activity. Furthermore, the absence of a direct comparison with established Alzheimer’s disease (AD) therapeutics in this study limits the contextualization of DAG-MAG-βHB’s efficacy within the broader landscape of AD treatments. Future research should include head-to-head evaluations with known compounds to benchmark its therapeutic potential, enabling a clearer understanding of its relative strengths and applicability. Additionally, while our findings in immortalized HMC3 cells and BBB models provide robust initial evidence for neuroprotection and inflammasome modulation, future studies should adopt complementary models to enhance the translational relevance of the results. Incorporating primary microglial cells or advanced organoid systems that replicate the native brain environment would address some limitations of immortalized cell lines, such as potential differences in gene expression and responsiveness. Furthermore, additional in vivo experiments, beyond the bioavailability and safety assessments conducted in this study, could provide deeper insights into the efficacy of DAG-MAG-βHB in mitigating neuroinflammation and cognitive decline in AD models. Expanding these approaches will help bridge the gap between preclinical findings and clinical applications, ultimately advancing the potential of DAG-MAG-βHB as a viable therapeutic strategy for AD.

## Figures and Tables

**Table 1:** Chemical composition of in vitro digestion fluids.

Constituent	Stock Solution		SSF	SGF	SIF
	mol/L	g/50 mL	mL		
**KCl**	0.5	1.86	10.79	4.60	6.18
**KH_2_PO_4_**	0.5	3.40	2.64	0.60	0.73
**NaHCO_3_**	1	4.20	4.86	8.33	38.64
**NaCl**	2	5.84	-	7.87	8.73
**MgCl_2_(H_2_O)_6_**	0.2	1.52	0.36	0.22	1
**(NH_4_)_2_CO_3_**	0.5	2.40	0.04	0.33	-

SSF, simulated salivary fluid; SGF, simulated gastric fluid; SIF, simulated intestinal fluid.

**Table 2:** Syber Green primers.

Primer	Sequence (5′-3′)	*Gene*
GAPDH-F	AAGGTGAAGGTCGGAGTCAAC	*GADPH*
GAPDH-R	GGGGTCATTGATGGCAACAATA	
NLRP3-F	GGAGAGACCTTTATGAGAAAGCAA	*NLRP3*
NLRP3-R	GCTGTCTTCCTGGCATATCACA	

## Data Availability

Data are available upon request.

## Supplementary Materials

The following supporting information can be downloaded at https://www.mdpi.com/article/10.3390/nu17010149/s1, Figure S1. ^1^H-NMR spectra of (a) DAG-ΒHB and (b) MAG-ΒHB. 13C-NMR spectra of Cc) DAG-ΒHB and d) MAG-ΒHB. Figure S2. ^1^H NMR COSY of (a) DAG-ΒHB and (b) MAG-ΒHB.
