# Supplementary material for: DAG-MAG-ΒHB: A Novel Ketone Diester Modulates NLRP3 Inflammasome Activation in Microglial Cells in Response to Beta-Amyloid and Low Glucose AD-like Conditions
Source: Nutrients. 2024 Dec 31;17(1):149. doi: 10.3390/nu17010149 (PMC11722608; doi:10.3390/nu17010149)

Figure S1. ¹H-NMR spectra of a) DAG-BHB and b) MAG-BHB. ¹³C-NMR spectra of c) DAG-BHB and d) MAG-BHB.

(a)

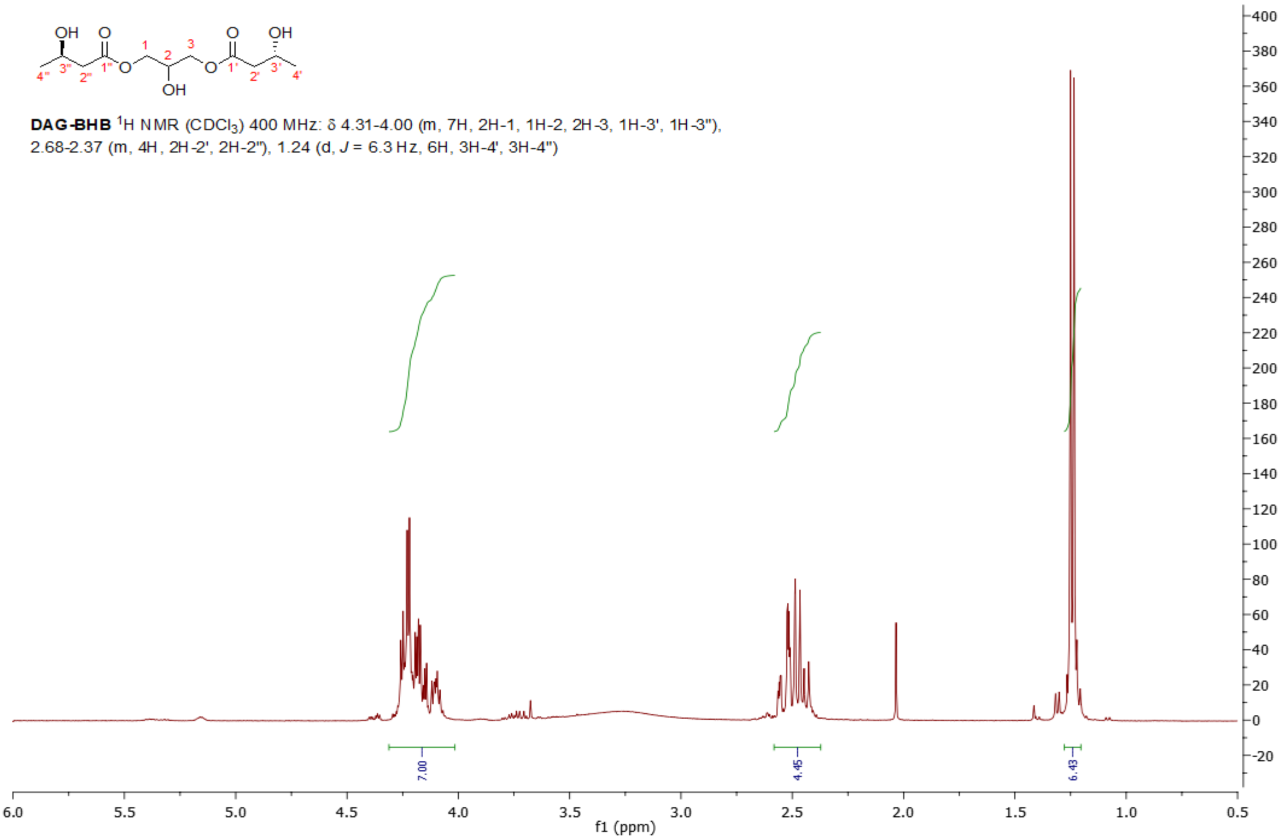

(b)

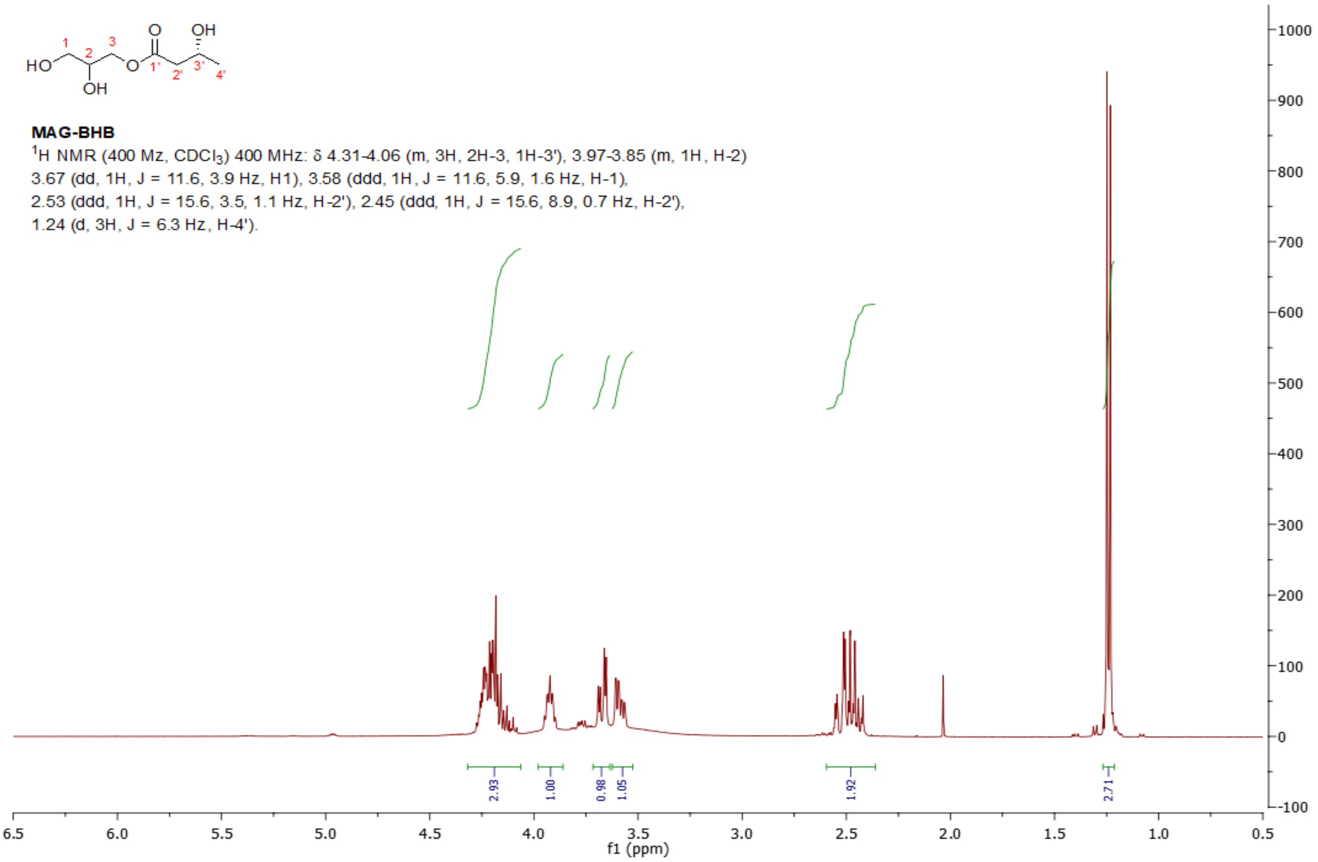

HO
OH
O
OH
MAG-BHB
1H NMR (400 Mz, CDCl3) 400 MHz: δ 4.31-4.06 (m, 3H, 2H-3, 1H-3'), 3.97-3.85 (m, 1H, H-2)
3.67 (dd, 1H, J = 11.6, 3.9 Hz, H1), 3.58 (ddd, 1H, J = 11.6, 5.9, 1.6 Hz, H-1),
2.53 (ddd, 1H, J = 15.6, 3.5, 1.1 Hz, H-2'), 2.45 (ddd, 1H, J = 15.6, 8.9, 0.7 Hz, H-2'),
1.24 (d, 3H, J = 6.3 Hz, H-4').
2.93
1.00
0.98
1.05
1.92
2.71
f1 (ppm)

(c)

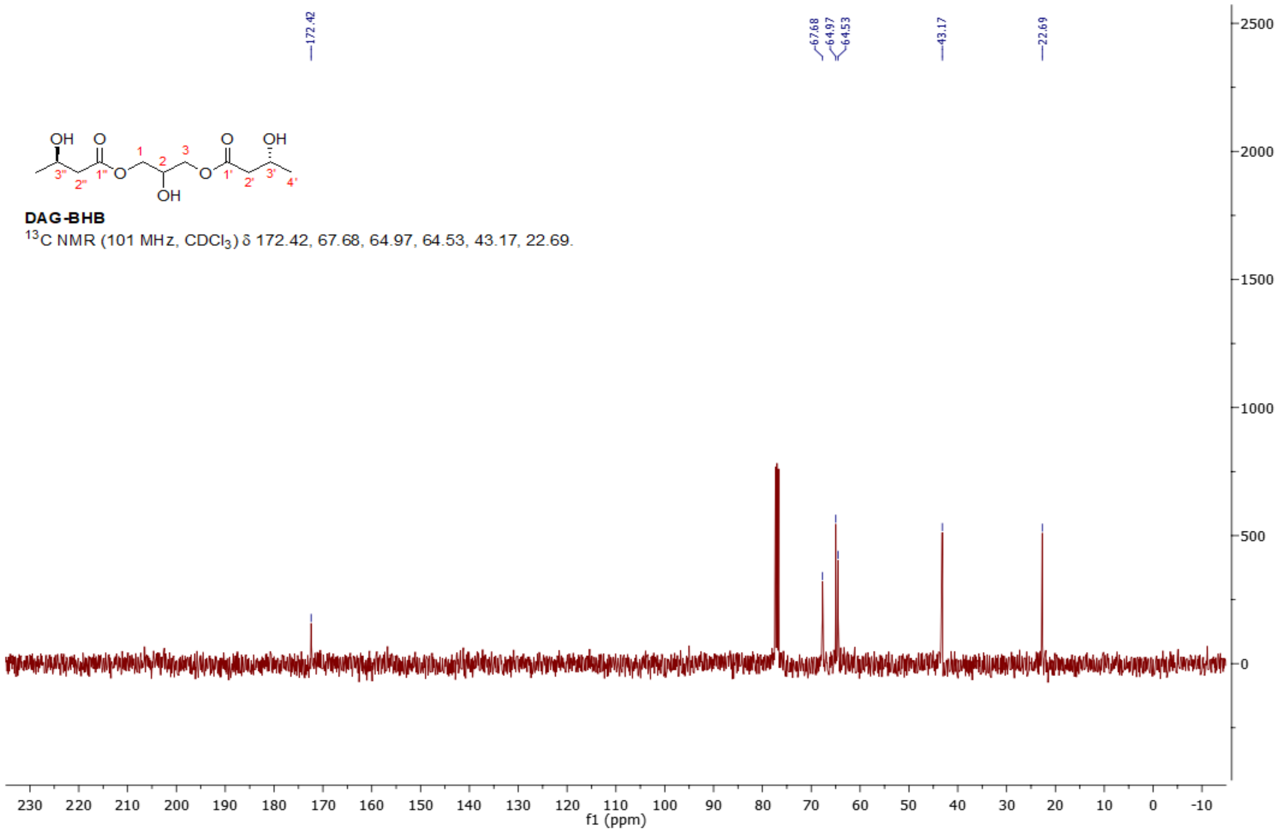

172.42
67.68
64.97
64.53
43.17
22.69
OH
O
O
OH
1
2
3
3''
2''
1''
1'
2'
3'
4'
OH
DAG-BHB
^{13}C NMR (101 MHz, CDCl$_3$) δ 172.42, 67.68, 64.97, 64.53, 43.17, 22.69.
2500
2000
1500
1000
500
0
230 220 210 200 190 180 170 160 150 140 130 120 110 100 90 80 70 60 50 40 30 20 10 0 -10
f1 (ppm)

(d)

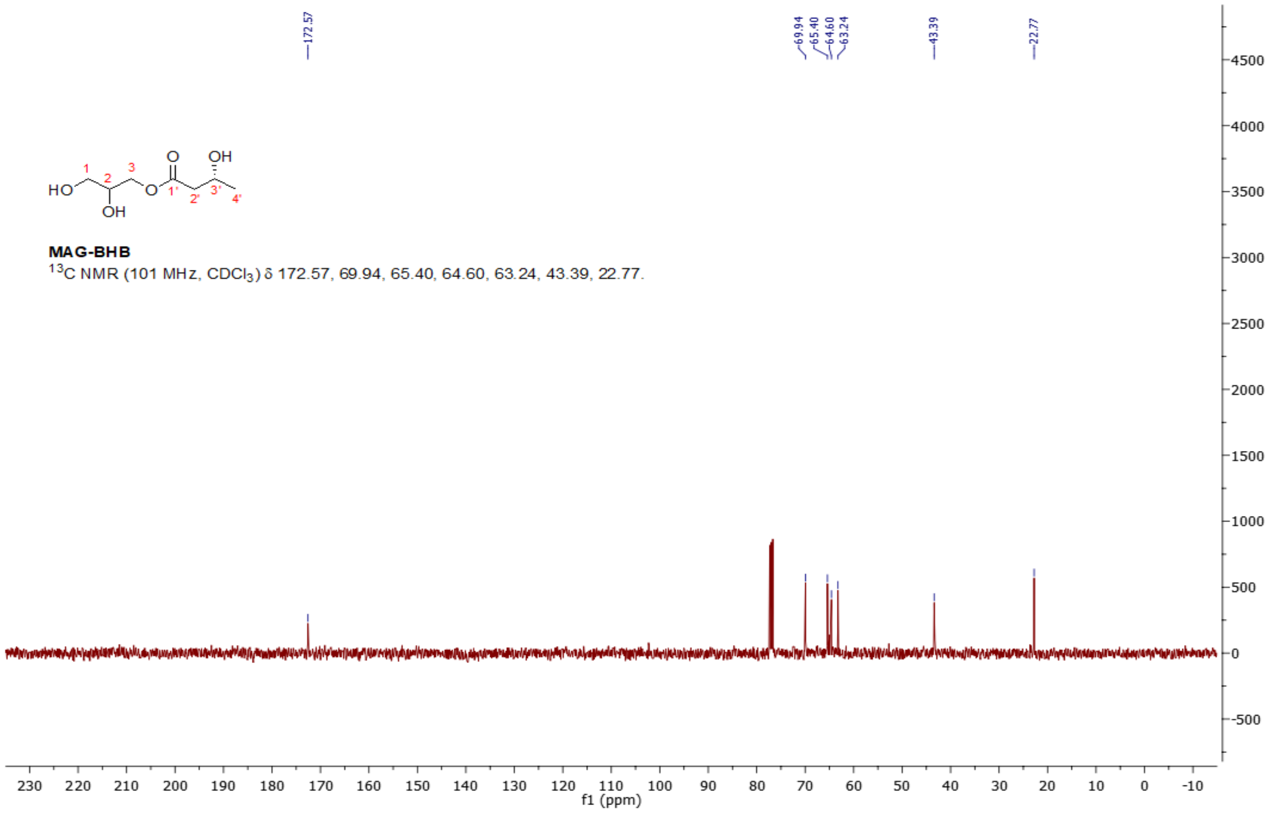

172.57
69.94
65.40
64.60
63.24
43.39
22.77
HO
OH
O
OH
1
2
3
1'
2'
3'
4'
MAG-BHB
13C NMR (101 MHz, CDCl3) δ 172.57, 69.94, 65.40, 64.60, 63.24, 43.39, 22.77.
f1 (ppm)

Figure S2. ^{1}H NMR COSY of a) DAG-BHB and b) MAG-BHB

(a)

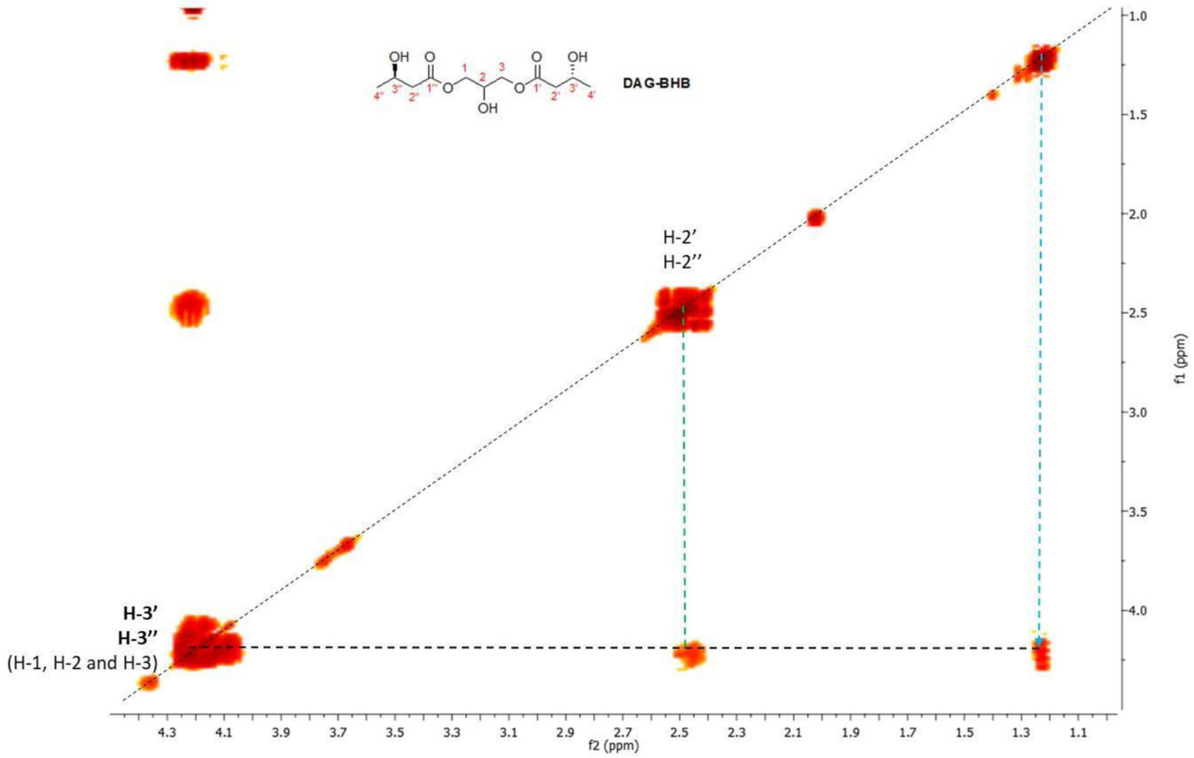

(b)

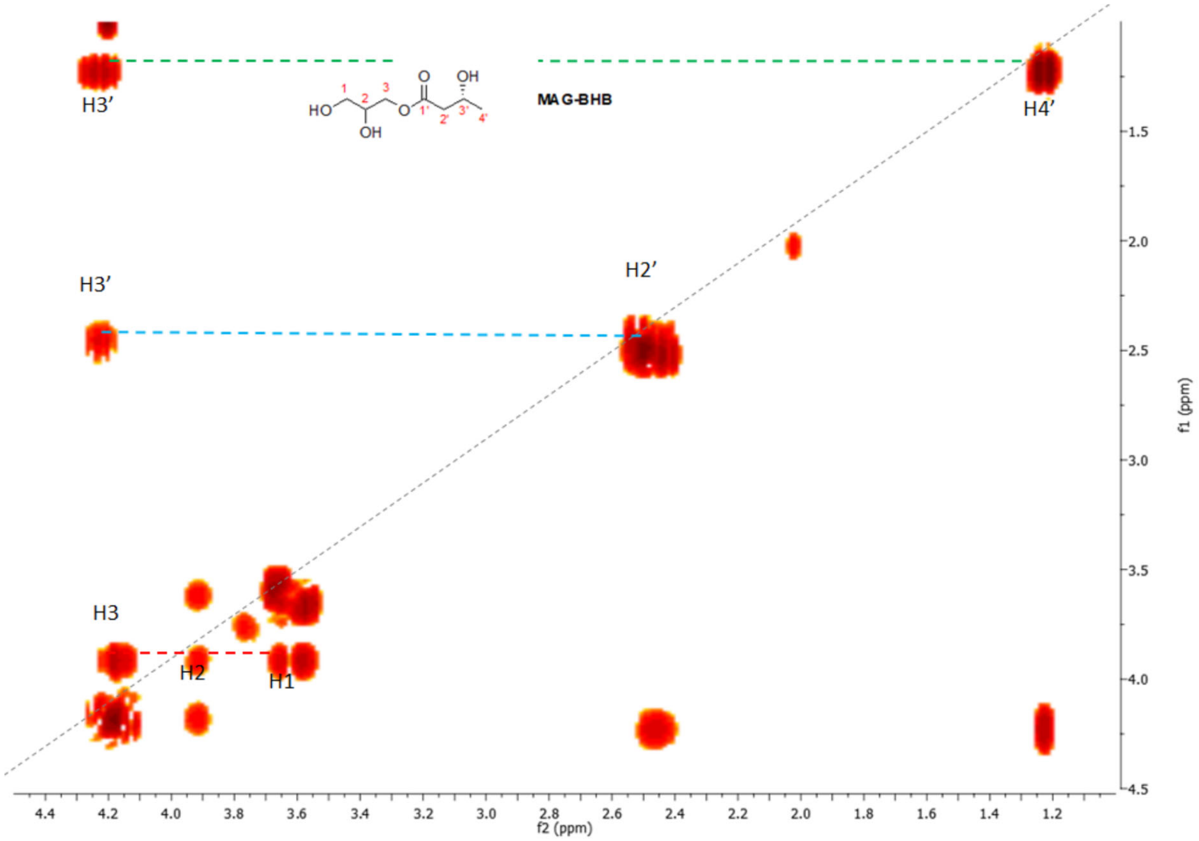

Supplement: Supplementary file 1 [file nutrients-17-00149-s001.zip › nutrients-3388240-supplementary.pdf]
